# Sporotrichosis in Amazon: series of 46 cases with emphasis on zoonotic transmission^☆^

**DOI:** 10.1016/j.abd.2025.501171

**Published:** 2025-08-09

**Authors:** Luiza Rennó Rocha de Oliveira, Isabela de Nazaré Tavares Cardoso Souza, Murilo dos Santos Souza, Carla Andrea Avelar Pires, Maria Amélia Lopes dos Santos, Francisca Regina Oliveira Carneiro

**Affiliations:** a Service of Dermatology, Universidade do Estado do Pará, Belém, PA,Brazil; b Universidade do Estado do Pará, Belém, PA, Brazil

Dear Editor,

Sporotrichosis is a chronic, granulomatous, subcutaneous fungal infection caused by fungi of the genus *Sporothrix*.^1^, ^2^ Transmission occurs mainly through direct contact with contaminated soil or organic matter^3^, ^4^ and through interaction with infected animals, especially cats.^1^ Sporotrichosis can manifest cutaneously or extracutaneously, mainly in immunocompromised patients.^5^

This is a descriptive and observational study carried out at the Dermatology Service of Universidade do Estado do Pará, a reference in secondary care for dermatological diseases in the North region, between April 2022 and June 2024, related to 46 patients with sporotrichosis. The research was approved by the Research Ethics Committee of the University (Opinion n. 5.647.696/2022). The present study followed the CARE checklist for case reports and series. Data collection was performed through medical records, including demographic information, clinical characteristics, and treatment details.

Of the 46 patients, there was a predominance of females (71.7%), probably due to the fact that women are usually the main caregivers of animals, increasing the risk of contamination. Age ranged from 6 to 85 years, with a mean of 41.3 years (Fig. 1). All participants were considered immunocompetent at the time of diagnosis.

**Figure 1:**
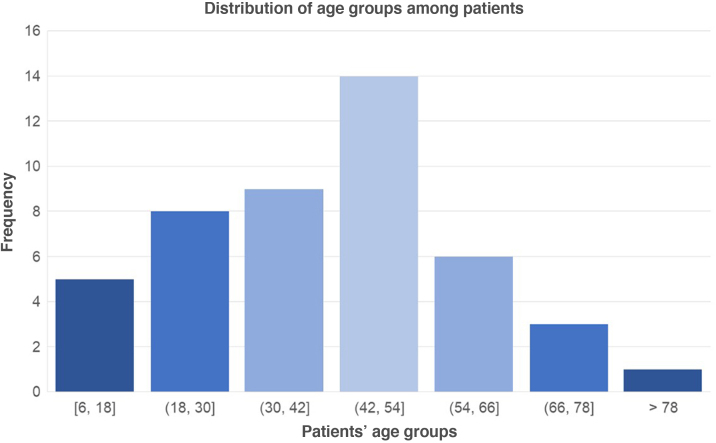
Distribution of age groups among patients diagnosed with sporotrichosis between April 2022 and June 2024 at a reference center in the Northern Region of Brazil.

Regarding epidemiology, 15.2% (n = 7) reported previous contact with soil or decomposing organic matter, while 95.5% (n = 44) reported contact with cats. Of these, 76% (n = 35) indicated recent scratches or bites, and 36.9% (n = 17) mentioned contact with cat secretions.

Histopathological examination was performed on 78.2% of the samples (n = 36), with reports suggestive or diagnostic of sporotrichosis. In 62.5% of the cases (n = 28), a culture was performed and confirmed in 32.6% (n = 15).

The skin lesions ranged from nodules to ulcers, with predominance in the extremities. In 28.3% of the cases (n = 13), concomitant involvement of lymphatic chains was observed. The localized cutaneous form was the most frequent one (69.5%), followed by the lymphocutaneous (28.3%; Fig. 2) and the severe/disseminated form (2.2%).

**Figure 2:**
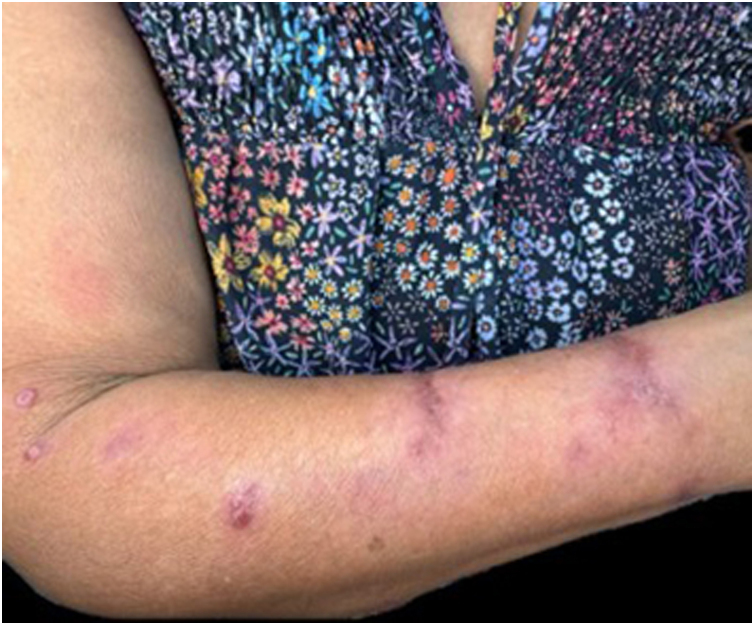
Patient with the lymphocutaneous form of sporotrichosis.

The evaluation of the animals showed that 63.6% of the felines (n = 28) had received a clinical veterinary diagnosis, and/or with the aid of direct mycological examination, of sporotrichosis. Information on clinical outcomes was obtained for 16 animals: 5 (31.2%) were euthanized due to the severity of the condition, 6 (37.5%) received successful treatment with itraconazole, and 5 (31.3%) died without veterinary intervention.

The medication prescribed in 93.5% of human cases (n = 43) was itraconazole, with doses ranging between 100 mg and 200 mg daily. In 4.5% of cases, potassium iodide (KI) was used due to the patients’ age (children, with doses between 1.4 and 2.1 g/day). Treatment lasted up to six months in 46% of patients, resulting in complete healing of the lesions. Adverse effects of itraconazole were reported by only three patients (6.9%) – myalgia, vertigo, requiring replacement by Terbinafine, with satisfactory response.

Regarding prognosis, 79% of cases (n = 36) showed healing of the lesions. Eight patients did not return for follow-up, and two are still being followed. In general, healing occurred between two and four months after the start of treatment. Outpatient discharge was recorded in 94.1% of cases, with complete healing and no recurrence or treatment failures.

Clinically, the most common form observed was the fixed or localized cutaneous form (Fig. 3), with predominant lesions being ulcers, nodules, and erythematous-infiltrated plaques. This difference in relation to other studies^6^ can be attributed to the higher number of immunocompetent patients in this study and early access to medical care. The diagnostic methods most often used in the present study were culture of the material and histopathological analysis, with culture on Sabouraud Dextrose Agar being considered the gold standard.^7^ Moreover, 12 of the 27 cases showed a negative culture, despite presenting suggestive histopathology. It is worth noting that some patients were already undergoing treatment at the time of diagnosis, which may have influenced the culture results.

**Figure 3:**
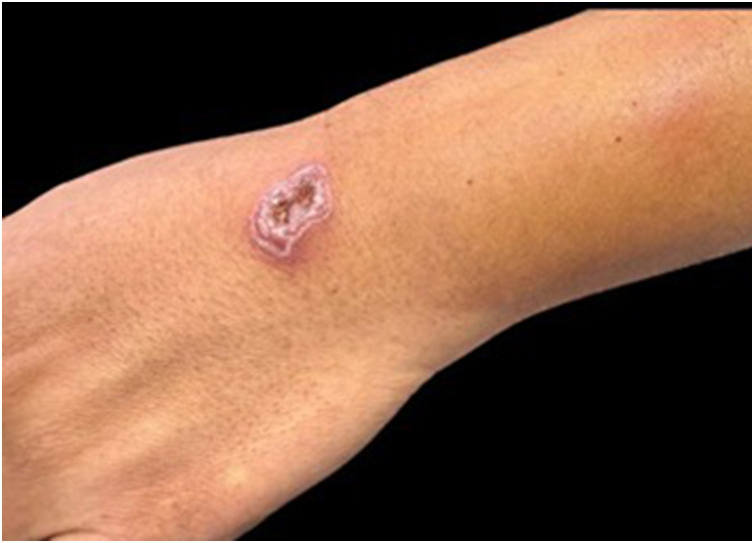
Patient with the fixed cutaneous form of sporotrichosis.

The results of this study (Table 1) highlighted the variety of clinical presentations and characteristics of the lesions, the good efficacy of the treatment used, the relatively short healing time (less than six months for 46% of patients), and the cure outcome. The positive therapeutic response to itraconazole in all cases reinforces the drug efficacy,^8^ already well established in national protocols.

**Table 1:** Clinical-epidemiological data of patients diagnosed with sporotrichosis between April 2022 and June 2024 from a reference center in the Northern Region of Brazil.

Clinical-epidemiological data		Total	(%)
Sex	Male	13	27,3%
	Female	33	71,7%
Origin	Metropolitan Region of Belém	2	4,3%
	Belém	44	95,7%
Feline Exposure	Yes	44	95,5%
	No	2	4,5%
Scratch, bite or contact with/secretions		37	84%
Diagnostic confirmation in the feline		28	63,6%
Feline under treatment		6	21,4%
Clinical form	Localized cutaneous	32	69,5%
	Lymphocutaneous	13	28,3%
	Severe/disseminated	1	2,2%
Medication	Itraconazole	43	93,5%
	Potassium Iodide	3	4,5%
Healing time after starting the treatment^a^	< 3 months	10	22%
	< 6 months	21	46%
	> 6 months	5	11%

a 8 patients lost to follow-up/2 patients still being followed.

Since the 1990s, interaction with infected felines has been widely associated with the increased incidence of cases in Brazil,^2^, ^9^ accounting for 95.5% of the reported causes of infection.^10^ This finding is corroborated by the data from the present study, which identified this route of transmission in 44 patients (95.5%).

Transmission occurs mainly through scratches, bites or contact with respiratory secretions from infected cats,^4^, ^9^ affecting especially veterinarians, owners, or animal rescue activists. Sanitary measures, veterinary control, and keeping felines in safe environments, without access to the street, are essential to reduce transmission between felines and humans.

The limitations of this research include the sample being restricted to a single center and the lack of prolonged follow-up of some patients. The need for accurate diagnostic methods and awareness of the disease is essential to prevent morbidity and sequelae. The lack of mandatory reporting in some states,^7^ such as Pará, and the lack of knowledge of the disease among physicians contribute to underreporting, impacting public control policies.

## Financial support

This study was funded by CNPq (*Conselho Nacional de Desenvolvimento Científico e Tecnológico*) and 10.13039/501100005288FAPESPA (*Fundação Amazônia de Amparo a Estudos e Pesquisas*) through PIBIC (*Programa Institucional de Bolsas de Iniciação Científica*) N. 026/2023 – Universidade do Estado do Pará (UEPA).

## Authors' contributions

Luiza Rennó Rocha de Oliveira: Collection of data, or analysis and interpretation of data; statistical analysis; drafting and editing of the manuscript or critical review of important intellectual content; collection, analysis and interpretation of data; critical review of the literature; approval of the final version of the manuscript.

Isabela de Nazaré Tavares Cardoso Souza: Collection of data, or analysis and interpretation of data; statistical analysis; drafting and editing of the manuscript or critical review of important intellectual content; collection, analysis and interpretation of data; critical review of the literature; approval of the final version of the manuscript.

Murilo dos Santos Souza: Collection of data, or analysis and interpretation of data; statistical analysis; drafting and editing of the manuscript or critical review of important intellectual content; collection, analysis and interpretation of data; critical review of the literature; approval of the final version of the manuscript.

Carla Andrea Avelar Pires: Design and planning of the study; effective participation in research orientation; intellectual participation in the propaedeutic and/or therapeutic conduct of the studied cases; critical review of the literature; approval of the final version of the manuscript.

Maria Amélia Lopes dos Santos: Design and planning of the study; effective participation in research orientation; intellectual participation in the propaedeutic and/or therapeutic conduct of the studied cases; critical review of the literature; approval of the final version of the manuscript.

Francisca Regina Oliveira Carneiro: Design and planning of the study; effective participation in research orientation; intellectual participation in the propaedeutic and/or therapeutic conduct of studied cases; critical review of the literature; approval of the final version of the manuscript.

## Conflicts of interest

None declared.
